# Supplementary material for: Exploring the causal role of the human gut microbiome in endometrial cancer: a Mendelian randomization approach
Source: Sci Rep. 2025 Apr 8;15:11953. doi: 10.1038/s41598-025-96740-x (PMC11978776; doi:10.1038/s41598-025-96740-x)
Supplement: Supplementary file 1 — Supplementary Material 1 [file 41598_2025_96740_MOESM1_ESM.pdf]

TITLE: **Exploring the causal role of the human gut microbiome in endometrial cancer: a Mendelian randomization approach**

AUTHORS: Ella Fryer, Charlie Hatcher, Rochelle Knight, Kaitlin H Wade

Supplementary Figures

Figure 1: Forward MR results for effect of the presence or absence of *G. unclassified, F. Erysipelotrichaceae* on endometrioid cancer risk using a more lenient p-value threshold.

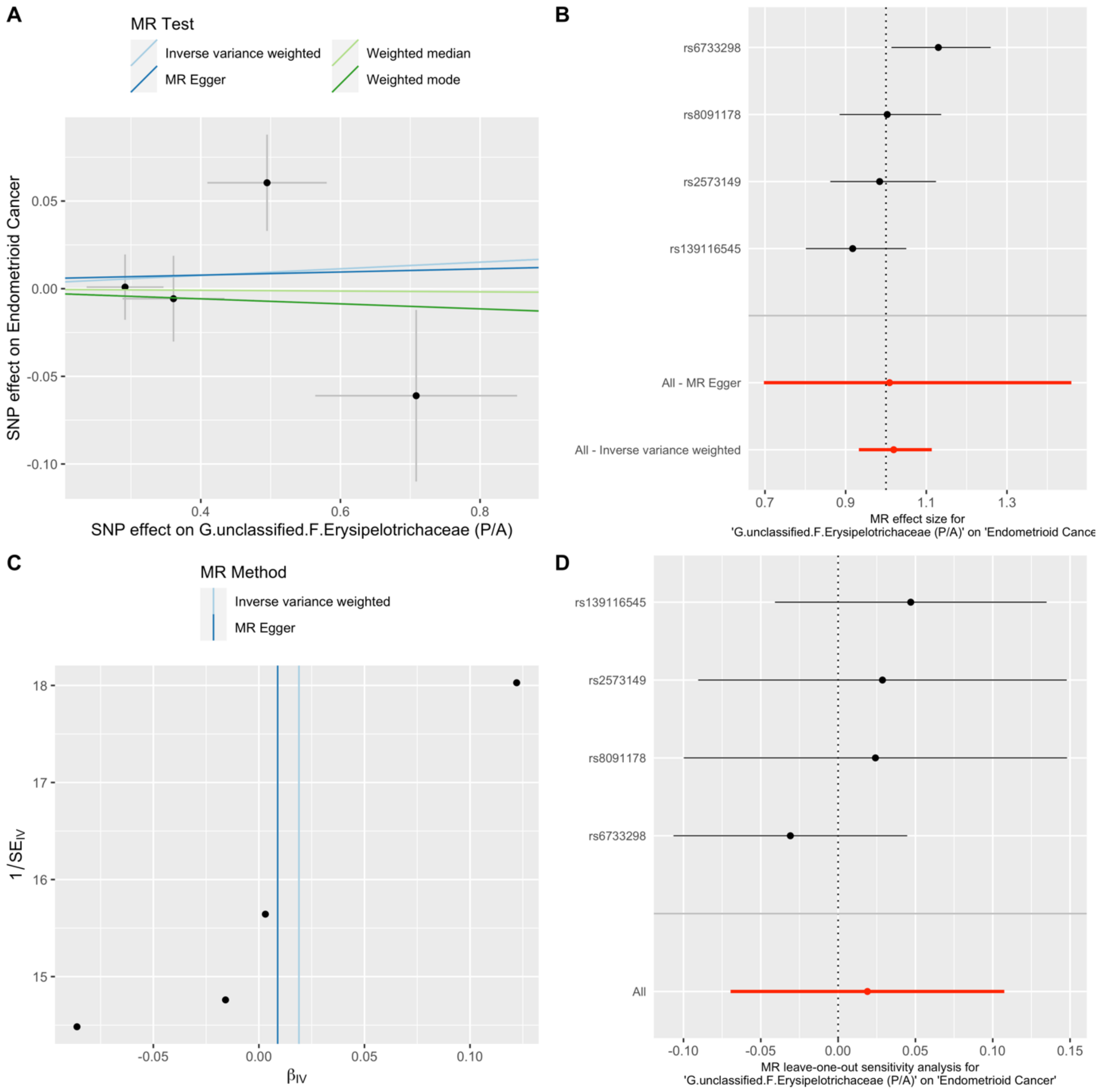

Abbreviations: MR = Mendelian randomization; SNP = single nucleotide polymorphism; P/A = presence versus absence. These plots were generated using the Two Sample MR package, showing the results of reverse MR analyses using a lenient p-value threshold (P<1x10-4) (enabling 4 SNPs to be used as instrumental variables for G. unclassified, F. Erysipelotrichaceae (P/A) and therefore pleiotropy-robust methods to be used) to test the

causal effect of the presence versus absence of this microbial trait on endometrioid cancer. A) scatter plot to compare all four methods; B) forest plot comparing effect of individual SNPs with the inverse variance weighted and MR Egger estimates; C) funnel plot to check for asymmetry; D) leave one out analysis to check if any one SNP is driving pleiotropy or asymmetry in the estimate.
